# Parenteral Nutrition and Intestinal Failure

**DOI:** 10.3390/nu9050466

**Published:** 2017-05-06

**Authors:** Barbara Bielawska, Johane P. Allard

**Affiliations:** 1 Department of Public Health Sciences, Queen’s University, Kingston, ON K7L 3N6, Canada; barbara.bielawska@queensu.ca; 2 Division of Gastroenterology, Department of Medicine, University of Toronto, Toronto, ON M5G 2C4, Canada; 3 Toronto General Hospital, University Health Network, Toronto, ON M5G 2C4, Canada

**Keywords:** parenteral nutrition, intestinal failure, home parenteral nutrition, short bowel syndrome

## Abstract

Severe short bowel syndrome (SBS) is a major cause of chronic (Type 3) intestinal failure (IF) where structural and functional changes contribute to malabsorption and risk of micronutrient deficiencies. Chronic IF may be reversible, depending on anatomy and intestinal adaptation, but most patients require long-term nutritional support, generally in the form of parenteral nutrition (PN). SBS management begins with dietary changes and pharmacologic therapies taking into account individual anatomy and physiology, but these are rarely sufficient to avoid PN. New hormonal therapies targeting intestinal adaptation hold promise. Surgical options for SBS including intestinal transplant are available, but have significant limitations. Home PN (HPN) is therefore the mainstay of treatment for severe SBS. HPN involves chronic administration of macronutrients, micronutrients, fluid, and electrolytes via central venous access in the patient’s home. HPN requires careful clinical and biochemical monitoring. Main complications of HPN are related to venous access (infection, thrombosis) and metabolic complications including intestinal failure associated liver disease (IFALD). Although HPN significantly impacts quality of life, outcomes are generally good and survival is mostly determined by the underlying disease. As chronic intestinal failure is a rare disease, registries are a promising strategy for studying HPN patients to improve outcomes.

## 1. Introduction

Intestinal failure (IF) is a state of “inability to maintain protein-energy, fluid, electrolyte or micronutrient balance” resulting from bowel resection or obstruction, dysmotility, congenital gastrointestinal defects, or loss of absorption as a consequence of disease [1]. Intestinal failure can be subdivided into three types. Type 1 IF is a transient state such as ileus following abdominal surgery, which may require a brief period of nutritional support, but is simple to manage and fully reverses without sequelae [2]. Type 2 IF occurs in the context of severe illness such as major surgery, where complications due to sepsis, metabolic derangement, and malnutrition require complex multidisciplinary care and specialized nutrition support including parenteral nutrition (PN) [2]. These patients may recover fully or progress to Type 3 intestinal failure, which is a chronic state of IF requiring long-term nutritional support, typically in the form of home parenteral nutrition (HPN) [3]. Type 3 IF may be reversible or irreversible [4]. While Type 3 IF often follows Type 2, it can also occur without surgery, such as in the case of motility disorders. The purpose of this review is to discuss HPN as a treatment for Type 3 IF, with a focus on short bowel syndrome.

## 2. Short Bowel Syndrome

When IF occurs in the context of bowel resection, congenital defects, or disease causing diffuse loss of absorptive surface, the condition is termed short bowel syndrome (SBS) [1]. SBS represents a spectrum, from mild dysfunction that can be overcome with pharmacologic and/or dietary measures with or without micronutrient supplementation, to a severe and disabling condition requiring intravenous fluids and electrolytes or PN. Severe SBS is by far the dominant entity encountered in Type 3 intestinal failure and requires HPN support.

### 2.1. Mechanisms of Malabsorption

**Figure 1:**
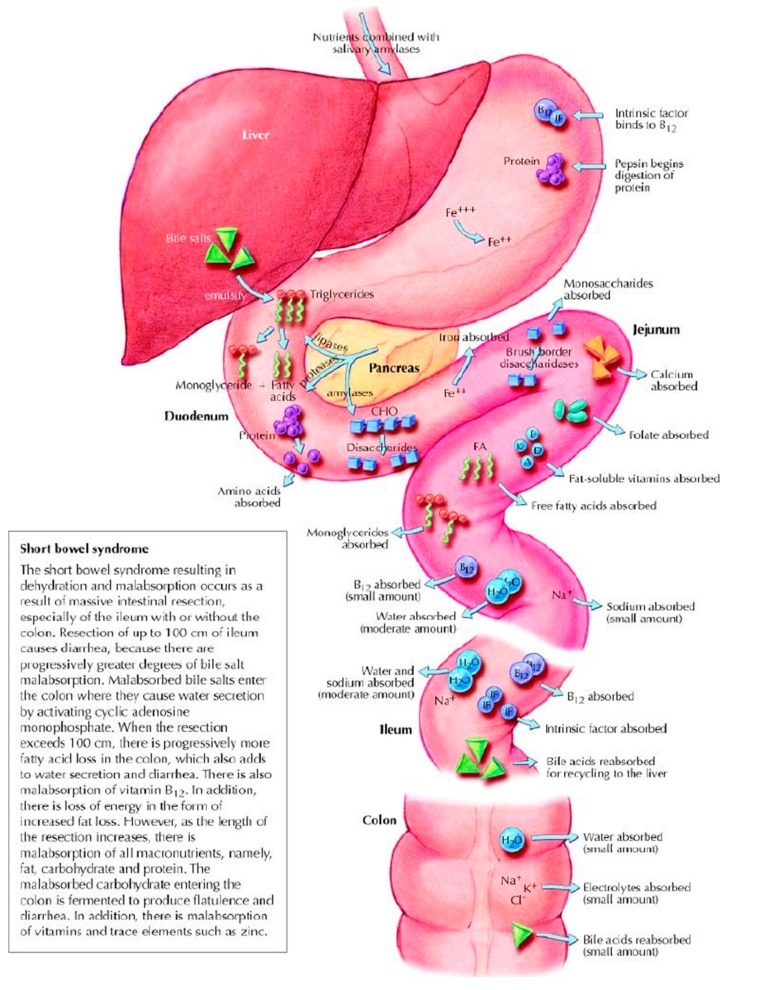
Normal absorption and digestion. Adapted with permission from Jeejeebhoy, K. N. (2002). Short bowel syndrome: A nutritional and medical approach. *CMAJ: Canadian Medical Association Journal*, *166*(10), 1297–1302.

The severity and type of malabsorption in SBS is determined by several factors. The greater the length of small bowel that is removed or rendered non-functional, the more absorptive surface area is lost. Mechanisms and locations of normal digestion and absorption of macro- and micronutrients are illustrated in Figure 1. Loss of ileum specifically results in malabsorption of vitamin B12, fat-soluble vitamins, and bile acids [5,6]. Loss of distal ileum and the ileocecal valve leads to rapid intestinal transit, gastric hypersecretion, and dumping due to loss of hormonal negative feedback signals, such as peptide YY [7]. Dumping and rapid transit result in inadequate mixing of nutrients with pancreaticobiliary secretions and insufficient mucosal contact for absorption to occur. Lack of normal motility and loss of ileocecal valve lead to small intestinal bacterial overgrowth (SIBO), which worsens malabsorption via direct nutrient consumption (such as B12) and bile acid deconjugation that leads to fat malabsorption [8]. SIBO generally produces symptoms such a bloating and diarrhea that may lead to reduced oral intake to alleviate symptoms. If the colon is removed or placed out of continuity with the small intestine, significant capacity for sodium and water absorption is lost [9], leading to dehydration, metabolic derangements, and renal failure. Furthermore, removal of the colon prevents salvage of calories from unabsorbed carbohydrates, which undergo fermentation by colonic bacteria to short chain fatty acids (SCFAs) that are absorbed in the colon [10].

In the case of massive resection, the intestine has a remarkable ability to adapt, and this is a chief determining factor for nutritional autonomy (independence from parenteral support). The process of intestinal adaptation can be broken down into three clinical phases [11]. The first phase after resection is characterized by profound gastric hypersecretion with high fluid and electrolyte loss. While most prominent in the first 1–2 months, hypersecretion can continue up to 6 months [1]. In the second phase, an adaptive response is seen and manifests as gradually improving absorption of micro and macronutrients, and consequently decreased losses of fluid. This phase is mediated by various intestinal hormones and growth factors, including growth hormone (GH), glucagon like peptide 2 (GLP-2), and epidermal growth factor (EGF), which promote structural and functional changes in the remnant small bowel as well as the colon to increase intestinal absorptive surface and absorptive capacity [11,12]. The maximum adaptation is typically achieved at 2 years, at which point the third phase of stabilization occurs and the adapted state is maintained [13].

### 2.2. Management

#### 2.2.1. Diet and Micronutrient Supplementation

Nutritional management of the patient with SBS requires understanding of physiology and consideration of individual anatomy and stage of adaptation. During the hypersecretory phase, fluid losses are typically the highest [14]. However, dehydration and salt depletion can be an issue at any phase, particularly in patients without a colon, and especially in the case of end-jejunostomy [12]. Patients are instructed to limit intake of simple sugars, eat small frequent meals, and to drink oral rehydration solutions rather than hypotonic liquids [15,16]. Parenteral fluids and electrolytes are often needed, but can be gradually weaned as adaptation progresses. Specific dietary recommendations depend on whether the patient has any colon in continuity with the small bowel, but generally all patients are encouraged to follow a hyperphagic (high calorie) diet with low or medium-fiber complex carbohydrates as the primary source of calories, followed by fat as tolerated [16]. Patients should have adequate protein intake, typically 20–30% of total calories [16]. Because the colon is the site of oxalate absorption, which is enhanced in the presence of steatorrhea, patients with colon in continuity must follow a low oxalate diet to reduce risk of nephrolithiasis [17]. Patients with symptoms of carbohydrate malabsorption, such as bloating and diarrhea, are typically instructed to follow a low lactose diet. However, lactose restriction need not be universal, as some patients do tolerate limited amounts [4]. In our practice, we discourage the use of fiber supplements to thicken ostomy effluent in patients with moderate to severe malabsorption, as these can increase stool output and impede contact of nutrients with intestinal mucosa [18]. Select patients may benefit from semi-elemental formulas or enteral feeding for supplemental calories.

All patients with chronic IF due to SBS require careful monitoring and replacement of micronutrients. While individualized supplementation is desirable, most providers use commercial preparations of trace elements to reduce costs [19]. Patients are particularly at risk for fat soluble vitamin (ADEK) deficiency, and this needs to be monitored regularly. In our practice, patients who do not receive daily parenteral vitamins (because they receive PN only a few days a week) are instructed to take a multivitamin, which typically maintains fat soluble vitamin sufficiency other than vitamin D, for which high dose additional supplementation is often required [20]. Those with significant ileal resection require lifelong parenteral B12 supplementation [5], which is typically administered as a once monthly intramuscular injection. In patients with voluminous diarrhea and chronic wounds, additional zinc supplementation may be required [21,22]. Calcium and iron are absorbed in the duodenum, which is rarely resected, and therefore typically do not require additional supplementation.

#### 2.2.2. Pharmacologic Therapy

Pharmacologic therapy is an essential adjunct to dietary measures and its principles are based on counteracting physiologic, functional, and anatomical changes of SBS. Antidiarrheal agents including codeine, loperamide, and diphenoxylate are used to reduce motility and increase transit time to facilitate absorption. These agents can be used alone or in combination, the latter being more effective [23]. Antisecretory therapy with H2 blockers or proton pump inhibitors (PPI) is most important in the first 6 months after resection to counter gastric hypersecretion and consequently diarrhea [24]. In patients with severe fluid losses refractory to combination antimotility agents and intravenous PPI, the somatostatin analogue octreotide may be used [25]. This can effectively reduce massive enteral fluid losses; however, its use should be reserved for the most refractory cases, as its inhibition of multiple gut hormones may impair intestinal adaptation [26]. Antibiotics are used in patients with suspected SIBO, typically in cyclical fashion [8].

The newest addition to pharmacologic management of SBS is the GLP-2 analogue teduglutide. GLP-2 is a small peptide released from enteroendocrine cells in the distal small intestine and proximal colon [11]. It enhances mucosal growth of the small intestine, increases mesenteric blood flow, improves gut barrier function, and may slow gastric motility and reduce secretion [27]. In patients with SBS, teduglutide leads to improvement in absorption of nutrients and fluids [28]. Teduglutide is currently approved for use in patients who have reached the third phase of intestinal adaptation and have stable ongoing requirements for parenteral support. In such patients, it has been shown to reduce the need for parenteral support in the form of reduction of PN volume and can lead to complete weaning of parenteral support in a small proportion of patients [29].

#### 2.2.3. Surgical Options

Surgical options for treatment of SBS include autologous gastrointestinal reconstruction and intestinal transplant. Autologous procedures typically involve intestinal lengthening for patients with dilated remnant small bowel or creation of a reversed intestinal segment when remnant bowel is non-dilated [30]. Intestinal lengthening procedures are typically reserved for pediatric patients [31] while creation of a reversed intestinal segment (to slow motility) remains somewhat experimental. All techniques carry significant risk of anastomotic breakdown, stricture, and potential injury to vasculature [32].

Intestinal transplant is in theory an excellent treatment option for SBS. However, high immunogenicity of the intestinal allograft remains a significant barrier in the long-term success of this strategy [33]. Enthusiasm for intestinal transplantation has recently waned, with decreasing case volumes worldwide [34]. Intestinal transplant is therefore reserved for the 10–15% of patients with chronic intestinal failure who fail PN due to complications [4].

## 3. Home Parenteral Nutrition

The aforementioned strategies are important components of SBS management, especially for milder cases. However, for the majority of patients with severe SBS, HPN is the cornerstone of therapy. Patients who require long-term PN cannot be kept in hospital indefinitely, and thus transition to HPN when they are clinically stable. There are four typical settings in which HPN is administered: IF due to surgical complications in the cool-down period prior to subsequent surgical intervention; IF due to cancer, where life expectancy is otherwise beyond that when death would occur from lack of nutrition (typically 3 months or greater); SBS following bowel resection where PN may be continued indefinitely or eventually weaned; and Type 3 IF due to miscellaneous causes such as dysmotility. There is significant global variation in the indications for HPN. In the UK, the main indications are Crohn’s disease, intestinal ischemia, and surgical complications [3]. Meanwhile, cancer is the dominant indication for HPN in the United States and Japan, making up approximately 40% of the HPN population in these countries [3]. In Canada, more than 90% of HPN use is in the context of SBS (32%), cancer (38%), and surgical complications (30%) [35]. 

### 3.1. HPN Initiation

HPN is typically managed by an interdisciplinary team, usually consisting of a physician, nurse specialist, dietician, and pharmacist who are trained in IF and PN. Patients are usually started on PN in hospital in the context of admission for acute illness (Type 2 IF) or due to nutritional or metabolic complications in Type 3 IF. In more stable patients who are not at risk for refeeding syndrome, PN may be initiated out of hospital with close monitoring.

PN initiation and management in the context of critical illness is reviewed elsewhere in this issue. In non-critically ill patients, PN initiation begins with a nutritional assessment to determine energy requirements. In the absence of indirect calorimetry, this is estimated using a simple weight-based calculation or by one of a number of formulas such as the Harris-Benedict or Mifflin St. Jeor equation [36]. Estimating equations perform reasonably well outside the context of extremes of weight, where they can be significantly inaccurate [37]. The simplest method of estimation uses patient weight in kilograms multiplied by desired kilocalories per day, with 25 kcal/kg/day being appropriate for most patients. In the presence of edema, usual body weight is used, while in the case of obesity (BMI ≥ 30 kg/m^2^), adjusted body weight is used [38]. In severe malnutrition, the calorie goal may be 30–35 kcal/kg/day [39]. In the presence of acute stress, the estimated requirements may be multiplied by a factor of 1.3–1.5. Use of a particular method of estimation has not been shown to improve patient outcomes and thus this process typically follows institutional preferences [39]. Patient weight is monitored and PN calories subsequently adjusted to target ideal body weight.

The next step of PN initiation involves partitioning the daily caloric requirements between the three types of macronutrients. First, protein requirement is calculated. In non-catabolic individuals with normal renal function, this is typically 0.8–1 g/kg/day [39]. This is increased to 1.5 g/kg/day or more in the presence of metabolic stress or conditions of active protein loss such as fistulas or protein-losing enteropathy [40]. After total protein dose is determined, the remaining non-protein calories are divided between carbohydrates (60–70%) and lipids (30–40%). Minimum lipid content is 1 g/kg/week in order to avoid essential fatty acid deficiency [4], while lipid content is typically kept below 1 g/kg/day to minimize the risk of liver complications (see Section 3.3.2). The total volume is set at approximately 35 mL/kg, but may vary widely depending on individual factors including daily fluid losses and comorbid conditions such as renal or heart failure [41].

Finally, electrolytes, trace elements, and vitamins are added to the solution. Electrolytes are added based on recommended daily intake and adjusted for individual requirements, including consideration of losses. Trace elements and multivitamins are available as commercially prepared mixtures, with compositions based on recommended daily requirements [39]. Micronutrients in PN are reviewed elsewhere in this issue. Additional medications may be added to the PN solution just prior to administration, including insulin, H2 receptor antagonists, heparin, and octreotide. At home, PN solutions are delivered to patients as ready-to-use bags or as three-in-one bags, where lipid, carbohydrate, and protein are separated in individual compartments and the partitions are broken to create a single chamber just before use [41].

Depending on the clinical situation and risk for refeeding syndrome, PN is initiated at the full or at partial requirement and then titrated to goal. Once the patient is clinically stable on the desired PN prescription, preparation for transition to HPN begins. This includes consideration of vascular access, cycling of the infusion, patient education, and logistical considerations for the patient home.

While short-term PN may be administered via a peripheral or central vein, HPN must be delivered by a central catheter. Options for access include peripherally inserted central catheters (PICC), tunneled subcutaneous catheters such as the Hickman, or a subcutaneously implanted venous port (Port-a-Cath). Choice of access often depends on the patient and institution, as well as local cost differences between access strategies. PICC lines are easiest to insert and remove, but have higher rates of local complications, malposition, and occlusion and may be preferred for situations where HPN will be required for less than 3–6 months [3,42,43]. Ports may be preferred for cosmetic reasons, but require needle puncture of the skin [43]. Regardless of catheter type, single lumen catheters are preferred due to lower risk of blood stream infection [44].

To maximize patient mobility and convenience at home, PN infusion time is minimized and the solution is infused overnight. PN infusion time can typically be reduced (cycled) to 10–15 h, depending on total volume and patient tolerance. The cycling is done gradually in hospital to ensure tolerance and safety. Longer infusion time is typically required in elderly patients, those with cardiac and renal comorbidities, and when total volume is very large such as during phase 1 of intestinal adaptation after surgery when gastrointestinal losses are high. The first and last 30 min of each infusion cycle involve gradual increase and decrease of infusion speed, respectively, to minimize metabolic disturbance such as dysglycemia and electrolyte imbalance [41]. Frequency of HPN administration is determined by individual energy, fluid, and electrolyte needs. Initially, patients tend to have daily infusions with progressive weaning of days as tolerated.

Depending on local practices and access to home care services, patients may be entirely responsible for administering their own PN at home or they may have significant home care assistance. Regardless, patient and caregiver education during the initiation process as well as on an ongoing basis is an essential component of successful and safe HPN [39]. Teaching is typically performed in multiple sessions and patients are discharged home only when they or their caregiver can demonstrate independent proficiency in all aspects of HPN delivery. This includes proper storage of PN solution bags, instillation of additives such as multivitamins or medications, operation and troubleshooting of infusion pump, as well as connecting and disconnecting from the pump. Patients must also receive specific teaching on care and access of their central vascular device. Patients receiving home care assistance require less specific knowledge, but must have sufficient education to address issues such as pump malfunction overnight when a nurse will not be present.

### 3.2. Monitoring

HPN requires regular clinical and laboratory monitoring. Patients are seen by a multidisciplinary nutrition team every 1–3 months at initiation, and eventually once a year when stable. The team assesses clinical symptoms, input and output, vital signs, and nutritional status including weight, BMI, and signs of nutritional deficiencies. In addition, the team monitors patients for potential PN-related complications such as central line infection or liver dysfunction. The PN prescription is then adjusted taking all of this into consideration along with laboratory findings.

The frequency of laboratory monitoring is based mostly on expert opinion [4]. After HPN initiation, electrolytes including Na^+^, K^+^, Cl^−^, HCO3^−^, as well as renal function and blood glucose should be measured frequently until stable, then at regular intervals. We typically obtain these every 1–2 weeks at the outset, followed by monthly and eventually every 3 months once stability is achieved. In addition, liver enzymes, bilirubin, albumin, Mg^+2^, Ca^+2^, and phosphate along with triglyceride and a complete blood count are measured every 3 months. Measurements of micronutrients including iron status, zinc, copper, B12, Vitamin A, Vitamin E, and Vitamin D, as well as international normalized ratio (INR) are performed annually in stable patients or more frequently according to the clinical situation. Bone density is measured at baseline and then every 1–2 years. New or unstable patients and those who undergo further intestinal resection are often instructed to measure their 24 h intake and output to adjust fluid and/or energy needs. In SBS patients with colon in continuity, we also obtain 24 h urine oxalate annually to biannually and use the results to reinforce the low oxalate diet. If urine oxalate remains high despite a low oxalate diet, calcium supplementation, often as calcium citrate, is added to meals to bind the oxalates and prevent colonic absorption. Calcium citrate may reduce the risk of developing kidney stones better than other forms of calcium (e.g., carbonate) in this setting [45]. Some will also consider reducing fat intake in the diet to decrease steatorrhea and hyperoxaluria, but this is not common practice as it will reduce oral energy intake and diet palatability, which may interfere with PN weaning during intestinal adaptation.

### 3.3. Complications of Long-Term PN

#### 3.3.1. Venous Access Related Complications

Problems related to central venous access are common in HPN and include blood stream infection, catheter malfunction, and thrombosis.

Catheter related blood stream infection (CRBSI) is a potentially serious complication of HPN therapy and is responsible for 70% of hospital admissions in HPN patients [41]. Incidence of CRBSI in the HPN population ranges between 0.35 to 2.27 per 1000 catheter days [46]. Risk factors include subcutaneous port rather than tunneled catheter, lack of patient training, frequent line access, opioid dependence, cancer, and presence of ostomy [47,48,49]. The most common organisms are Gram positive bacteria, which are typically coagulase negative staphylococci [48]. Fungal organisms represent approximately 10% of cases [48,49]. Diagnosis is typically made by blood culture and treatment includes antimicrobial therapy and consideration of catheter removal if necessary.

With appropriate antimicrobial therapy, catheter removal may be avoided in approximately 70% of cases [46]. Catheter removal is typically indicated for fungal infections, concurrent catheter dysfunction, tunnel infection, and life threatening sepsis [46]. It is worth noting that a small proportion of patients are responsible for the majority of CRBSI, with 12% of patients accounting for 75% of CRBSI in one large study [46]. Aseptic protocols and good catheter care have been shown to decrease risk of CRBSI [46]. In cases of recurrent infection, antibiotic locks such as with taurolidine appear to be highly effective [50], but are not a substitute for good catheter care.

Other less common infectious complications related to vascular access are exit site infection and infection of the catheter tunnel. Exit site infection may be treated with systemic antibiotics, while infection of the tunnel is an indication for line removal [3].

Catheter occlusion, displacement, and fracture are much less common than CRBSI. Occlusion may occur due to fibrin deposits or deposition of lipids from PN. Good catheter care including flushing the line reduces lipid deposits, while fibrin clots may be treated with instillation of fibrinolytic such as tissue plasminogen activator [3]. Displacement and fracture require line change. These are mostly seen with PICC lines and limit their long-term use [42]. 

Catheter related venous thrombosis is an important complication that can lead to loss of vascular access. The incidence has been reported as 0.027 episodes per catheter year [51]. Risk factors include pro-thrombotic conditions such as cancer, catheter trauma, and sepsis [52]. In patients with SBS, absorption of oral anticoagulants is variable and thus subcutaneous injection of low molecular weight heparin may be preferable for both prophylactic and therapeutic anticoagulation [4]. Use of novel anticoagulants has been reported in SBS [53], but there is insufficient evidence to date to advocate their widespread use in this setting.

#### 3.3.2. Intestinal Failure Associated Liver Disease (IFALD)

IFALD is a common and important concern in HPN and remains a major cause of morbidity and mortality in this population. Mild abnormalities in hepatic biochemistry are very common, seen in 30–40% of HPN patients [43]. While older series reported incidence of end stage liver disease between 15–40%, more recent studies have demonstrated this to be much lower [54], possibly due to improvements in HPN care including introduction of new types of lipid emulsions. However, the risk of significant liver disease increases with longer duration of HPN [55,56].

IFALD has mostly replaced the term PN-associated liver disease (PNALD) and encompasses the myriad of nutrient and non-nutrient factors that contribute to liver dysfunction in this setting. There is no unifying theory for IFALD [55]. Non-nutrient factors include hepatotoxic medications, endotoxemia related to sepsis and bacterial overgrowth, and intrinsic liver disease [3]. Cholelithiasis is common in the HPN setting and is related to lack of oral intake-induced gallbladder stimulation. This may lead to biliary obstruction in a small proportion of patients. A French group found a 7.6% incidence of biliary complications in 2 years with 38% of HPN patients developing cholelithiasis in that time period [57].

Both nutrient toxicity and deficiency are postulated to contribute to IFALD. Overfeeding with lipids (especially omega 6 fatty acids above 1 g/kg/day) [56] or carbohydrates [58], phytosterols in lipid emulsions [59], aluminum [60], copper [61], and manganese [62] have been associated with liver dysfunction. Deficiencies in essential fatty acids, choline [63], taurine [64], and carnitine have also been linked to IFALD [3]. The intestinal microbiome [65] and SIBO have also been implicated in IFALD [66]. Pathologically, IFALD manifests as steatosis (micro or macrovesicular), steatohepatitis, and intrahepatic cholestasis [43].

Risk of IFALD may be reduced by limiting lipid dose to 1 g/kg/day and using lipid emulsions that are not 100% soybean oil based [41]. Enteral feeding is also protective [67], likely via stimulation of bile flow. Cycling of TPN (versus continuous infusion) is protective [68]. If liver dysfunction is progressive, lipid dose may be reduced or lipids held temporarily, or patients may be switched to an alternate lipid emulsion [4]. Ursodeoxycholic acid can improve cholestasis [69]. Treatment of SIBO may be of benefit [66]. Overt or impending liver failure due to IFALD is an indication for small bowel transplant [33].

#### 3.3.3. Other Metabolic Complications

Patients with SBS who require PN are at risk of chronic dehydration and consequent renal failure [70], as well as nephrolithiasis. Nephrolithiasis may be related to dehydration as well as hyperoxaluria in the presence of colon in continuity [71]. Patients should have urine output of at least 1 L per day and follow a low oxalate diet where applicable [17]. Supplemental parenteral fluids such as normal saline beyond that provided by PN bags are required to achieve adequate urine output in patients with high volume losses.

Patients are at risk for micronutrient deficiency or excess, and this must be monitored regularly, as outlined above.

Metabolic bone disease is a prominent complication of HPN and is discussed elsewhere in this journal issue.

### 3.4. Outcomes of Home Parenteral Nutrition

Chronic intestinal failure is potentially reversible, as 20–50% of patients who require HPN are eventually weaned off PN [4,72]. Consequently, delivery of HPN requires ongoing attention to individual nutritional as well as fluid and electrolyte needs, with prompt adjustments in the face of changes in clinical status. Using the pharmacologic and dietary strategies outlined above, the goal is to minimize parenteral requirements. This process of weaning PN is complex and highly individualized to each patient’s unique situation, and is thus beyond the scope of this article. In the case of SBS after major intestinal resection, PN may be gradually weaned as intestinal adaptation progresses. When the length of the remaining small intestine is below 150 cm, the risk of permanent intestinal failure increases. Permanent IF is typical for 100 cm or less of small intestine with end enterostomy, 65 cm of jejunum with jejunocolic anastomosis, and 35 cm of small bowel in a jejuno-ileo-colic configuration [13,43]. More than 90% of patients who cannot be weaned from PN within 2 years of surgery remain PN dependent [13]. 

HPN is associated with reduced quality of life (QOL) compared to the general population, and has previously been found to be comparable to that of patients on chronic hemodialysis [73]. More recent data suggest this impairment is not as severe [74]. Factors associated with reduced quality of life in HPN include younger age, chronic narcotic use, nocturia, and greater number of infusions per week [3,75]. In patients with severe malnutrition, HPN is associated with improvement in QOL [76]. Presence of ostomy is associated with significant reduction in QOL in HPN patients and this demonstrates improvement if ostomy can be reversed [75]. Technological advances such as portable infusion pump rather than pole-mounted pump have also been demonstrated to improve QOL in HPN patients [77]. QOL also tends to improve with longer duration on HPN [78].

Survival in HPN patients is typically determined by the underlying disease. Patients with inflammatory bowel disease have 5 year survival in excess of 90% on HPN, while those with motility disorders and cancer have the poorest survival [79]. Death related to HPN therapy is rare [80]. 

Failure of HPN is said to exist when patients have impending or overt liver failure secondary to PN, loss of central venous access (thrombosis of 2 or more central veins), frequent severe CRBSI, and frequent and severe dehydration despite HPN and supplemental intravenous hydration [81]. Intestinal transplant is indicated in these scenarios. 

### 3.5. The Future of HPN Research

Chronic intestinal failure requiring HPN is a very rare entity. In Europe, the prevalence of benign Type 3 IF requiring HPN is 5 to 20 per million [4]. The majority of HPN literature to date has been based on single institutional experiences and surveys. Creation of registries is a promising strategy to combine data from multiple centers in order to study this rare entity.

The Canadian Home Total Parenteral Nutrition registry was established in 2006 with the goal of collecting robust data for the purpose of quality assurance, developing best practice guidelines, and for clinical outcomes research. Eight HPN programs in six provinces across the country contribute to the registry, which now includes data on more than 500 patients. The database prospectively collects information on demographics as well as comprehensive laboratory and clinical data that include information regarding medical history, anatomy, HPN indications, prescriptions, and complications. Data are collected at baseline, every 2 years, and at HPN discontinuation or death.

To date, the database has produced publications on the topics of metabolic bone disease [82], CRBSI [83], vitamin K supplementation in relation to bone mineral density [84], HPN use in systemic sclerosis [85], manganese toxicity [86], and trace element prescription [19]. The database has also been used to study changes in HPN practice and demography [35]. In Canada, these studies have led to improvements in clinical practice and outcomes, such as reducing the incidence of central line infection [35], with workshops reviewing data from HPN programs organized during the Canadian Nutrition Society annual meetings. Therefore, in rare diseases such as SBS and HPN, a registry is very useful to track patients and improve management. 

## 4. Conclusions

In patients with chronic intestinal failure who cannot be managed with pharmacologic and dietary therapy alone, home parenteral nutrition is an effective long-term treatment, albeit complex and requiring attentive multidisciplinary care. New therapies aimed at promoting intestinal adaptation hold promise for reducing HPN burden in patients with benign IF.
